# Ethyl 2-(3,5-dinitro­benzamido)­benzoate

**DOI:** 10.1107/S1600536811053074

**Published:** 2011-12-14

**Authors:** Sohail Saeed, Naghmana Rashid, Rizwan Hussain, Wing-Tak Wong

**Affiliations:** a Department of Chemistry, Research Complex, Allama Iqbal Open Unicversity, Islamabad 44000, Pakistan; b National Engineering & Scientific Commission, PO Box 2801, Islamabad, Pakistan; c Department of Chemistry, The University of Hong Kong, Pokfulam Road, Pokfulam, Hong Kong SAR, People’s Republic of China

## Abstract

The title mol­ecule, C_16_H_13_N_3_O_7_, is slightly twisted, with the dihedral angle between the two benzene ring planes being 17.4 (1)°. An intra­molecular N—H⋯O hydrogen bond is observed. In the crystal, weak C—H⋯O hydrogen bonds link the mol­ecules into chains along the *b* axis.

## Related literature

For background to the biological activity of *N*-substituted benzamides and their use in synthesis, see: Saeed *et al.* (2011*a* ▶,*b* ▶). For the structures of related chloro­phenyl­benzamides, see: Gowda *et al.* (2007*a* ▶,*b* ▶,*c* ▶). For hydrogen-bond motifs, see: Bernstein *et al.* (1995 ▶). For bond-length data, see: Allen *et al.* (1987 ▶). For *ortho*-hydrogen steric hindrance, see: Karle & Brockway (1944 ▶).
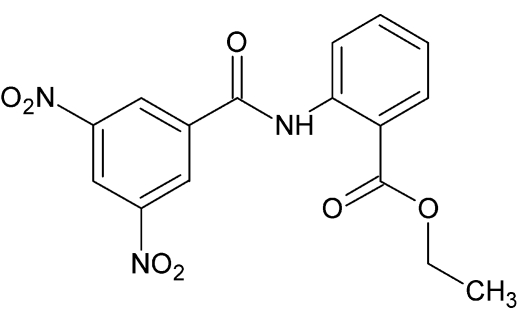


## Experimental

### 

#### Crystal data

C_16_H_13_N_3_O_7_
*M* *_r_* = 359.29
Monoclinic, 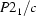

*a* = 12.4662 (4) Å
*b* = 17.7213 (5) Å
*c* = 7.4352 (2) Å
β = 96.658 (2)°
*V* = 1631.49 (8) Å^3^
*Z* = 4
Mo *K*α radiation
μ = 0.12 mm^−1^
*T* = 296 K
0.52 × 0.30 × 0.26 mm

#### Data collection

Bruker APEXII CCD diffractometer
Absorption correction: multi-scan (*SADABS*; Sheldrick, 2004 ▶) *T*_min_ = 0.942, *T*_max_ = 0.970
16092 measured reflections
2808 independent reflections
1961 reflections with *I* > 2σ(*I*)
*R*_int_ = 0.040

#### Refinement

*R*[*F* ^2^ > 2σ(*F* ^2^)] = 0.049
*wR*(*F* ^2^) = 0.174
*S* = 1.11
2808 reflections
237 parameters
H-atom parameters constrained
Δρ_max_ = 0.18 e Å^−3^
Δρ_min_ = −0.22 e Å^−3^

### 

Data collection: *APEX2* (Bruker, 2007 ▶); cell refinement: *SAINT* (Bruker, 2007 ▶); data reduction: *SAINT*; program(s) used to solve structure: *SHELXS97* (Sheldrick, 2008 ▶); program(s) used to refine structure: *SHELXL97* (Sheldrick, 2008 ▶); molecular graphics: *Mercury* (Macrae *et al.*, 2008 ▶); software used to prepare material for publication: *SHELXL97*.

## Supplementary Material

Crystal structure: contains datablock(s) global, I. DOI: 10.1107/S1600536811053074/fk2047sup1.cif

Structure factors: contains datablock(s) I. DOI: 10.1107/S1600536811053074/fk2047Isup2.hkl

Additional supplementary materials:  crystallographic information; 3D view; checkCIF report

## Figures and Tables

**Table 1:** Hydrogen-bond geometry (Å, °)

*D*—H⋯*A*	*D*—H	H⋯*A*	*D*⋯*A*	*D*—H⋯*A*
N1—H1⋯O2	0.86	1.92	2.641 (3)	140
C16—H16*A*⋯O3^i^	0.96	2.55	3.402 (4)	148

Symmetry code: (i) 
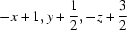
.

**Fig. 1.**
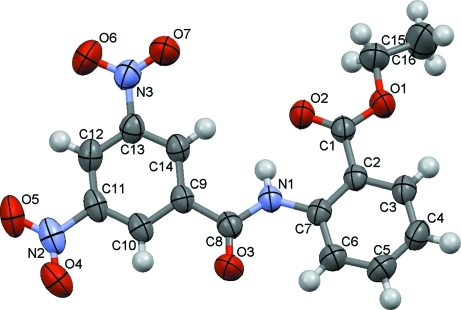
Molecular structure of the title compound with displacement ellipsoids at the 50% probability level.

**Fig. 2.**
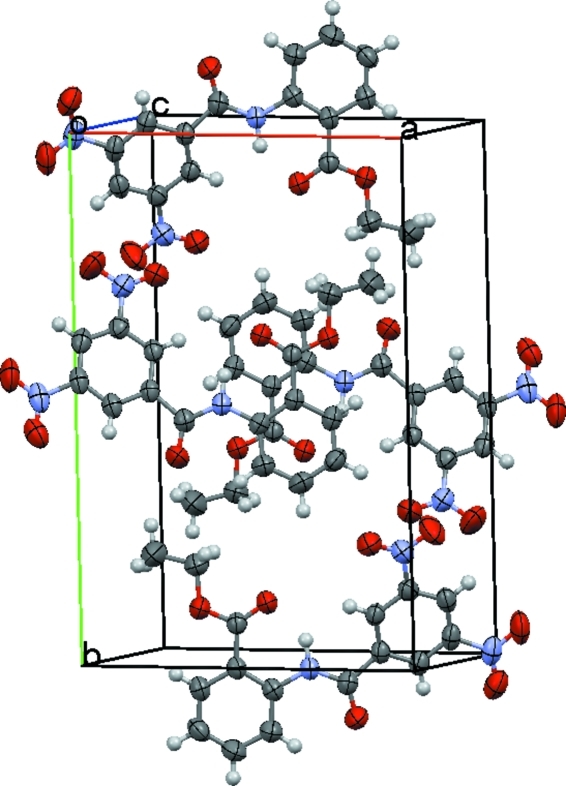
The packing diagram of the compound projected along the c-axis.

## supplementary crystallographic information

### Comment

In spite of the fact that the molecule is an extensively conjugated aromatic system, the molecule is not co-planar. This may be due to the steric hindrance between the *ortho*-H ··· amide H-atoms. The twisting away from coplanarity may help to relief this steric hindrance and results in an H14···H1 distance of 2.016 Å. This is in analogy to Karle and Brockway's suggestion that the steric hindrance between the *ortho* hydrogen atoms in biphenyl may be the reason for the non-coplanarity of the structure (Karle and Brockway, 1944). The dihedral angle between the two phenyl ring planes is about 17.4 (1)°. Both nitro groups are slightly twisted, 4.9 (2)° and 4.0 (2)° respectively, from the phenyl ring plane, C9—C11.

There is an intra-molecular N1—H1···O2 interaction. A weak intermolecular C16—H16A···O3(1 - *x*,1/2 + *y*,3/2 - *z*) hydrogen bond may help to align the molecules to endless chains along the *b*-axis in the crystal lattice. In addition, the conjugated ring planes of the title molecules are stacked along the *c*-axis with perpendicular distance between ring planes being 3.38 (1) Å.

### Experimental

To a 250 ml round flask fitted with a condenser ethyl *ortho*-amino benzoate (0.1 mol), dichloromethane (15 ml) and triethylamine (0.5 ml) was added under stirring. 3,5-dinitroenzoyl chloride (0.1 mol) was added gradually. The reaction mixture was stirred at room temperature for 1 h and then refluxed for 2 h. The product precipitated as a colourless powder, which was washed three times with water and dichloromethane. Recrystallization from ethyl acetate produced the crystals of the title compound.

### Refinement

The structure was solved by direct methods (*SHELXS97*, Sheldrick, 2008) and expanded using Fourier techniques. All non-H atoms were refined anisotropically.

All H atoms are observable from difference Fourier map but were refined riding at idealized geometrical positions with C—H = 0.93, 0.96 and 0.97Å for phenyl, methyl and methylene H-atoms and N—H = 0.86 Å and *U*_iso_(H) = 1.2*U*_eq_(C / N) and *U*_iso_(H) = 1.5*U*_eq_(C-methyl).

### Crystal data

**Table:**

C_16_H_13_N_3_O_7_	*F*(000) = 744
*M**_r_* = 359.29	*D*_x_ = 1.463 Mg m^−^^3^
Monoclinic, *P*2_1_/*c*	Mo *K*α radiation, λ = 0.71073 Å
Hall symbol: -P 2ybc	Cell parameters from 16092 reflections
*a* = 12.4662 (4) Å	θ = 2.8–25.0°
*b* = 17.7213 (5) Å	µ = 0.12 mm^−^^1^
*c* = 7.4352 (2) Å	*T* = 296 K
β = 96.658 (2)°	Block, colourless
*V* = 1631.49 (8) Å^3^	0.52 × 0.30 × 0.26 mm
*Z* = 4	

### Data collection

**Table:**

Bruker APEXII CCD diffractometer	2808 independent reflections
Radiation source: fine-focus sealed tube	1961 reflections with *I* > 2σ(*I*)
graphite	*R*_int_ = 0.040
ω and φ scan	θ_max_ = 25.0°, θ_min_ = 2.8°
Absorption correction: multi-scan (*SADABS*; Sheldrick, 2004)	*h* = −14→14
*T*_min_ = 0.942, *T*_max_ = 0.970	*k* = −21→21
16092 measured reflections	*l* = −8→8

### Refinement

**Table:**

Refinement on *F*^2^	Secondary atom site location: difference Fourier map
Least-squares matrix: full	Hydrogen site location: inferred from neighbouring sites
*R*[*F*^2^ > 2σ(*F*^2^)] = 0.049	H-atom parameters constrained
*wR*(*F*^2^) = 0.174	*w* = 1/[σ^2^(*F*_o_^2^) + (0.0679*P*)^2^ + 0.9448*P*] where *P* = (*F*_o_^2^ + 2*F*_c_^2^)/3
*S* = 1.11	(Δ/σ)_max_ < 0.001
2808 reflections	Δρ_max_ = 0.18 e Å^−^^3^
237 parameters	Δρ_min_ = −0.22 e Å^−^^3^
0 restraints	Extinction correction: *SHELXL97* (Sheldrick, 2008), Fc^*^=kFc[1+0.001xFc^2^λ^3^/sin(2θ)]^-1/4^
Primary atom site location: structure-invariant direct methods	Extinction coefficient: 0.013 (2)

### Special details

**Table:**

Geometry. All e.s.d.'s (except the e.s.d. in the dihedral angle between two l.s. planes) are estimated using the full covariance matrix. The cell e.s.d.'s are taken into account individually in the estimation of e.s.d.'s in distances, angles and torsion angles; correlations between e.s.d.'s in cell parameters are only used when they are defined by crystal symmetry. An approximate (isotropic) treatment of cell e.s.d.'s is used for estimating e.s.d.'s involving l.s. planes.
Refinement. Refinement of *F*^2^ against ALL reflections. The weighted *R*-factor *wR* and goodness of fit *S* are based on *F*^2^, conventional *R*-factors *R* are based on *F*, with *F* set to zero for negative *F*^2^. The threshold expression of *F*^2^ > σ(*F*^2^) is used only for calculating *R*-factors(gt) *etc*. and is not relevant to the choice of reflections for refinement. *R*-factors based on *F*^2^ are statistically about twice as large as those based on *F*, and *R*- factors based on ALL data will be even larger.

### Fractional atomic coordinates and isotropic or equivalent isotropic displacement parameters (Å^2^)

**Table:**

	*x*	*y*	*z*	*U*_iso_*/*U*_eq_	
O1	0.66429 (15)	0.10807 (11)	0.9056 (3)	0.0724 (6)	
O2	0.49758 (15)	0.11624 (11)	0.7638 (3)	0.0678 (6)	
O3	0.29471 (18)	−0.10623 (12)	0.5275 (3)	0.0855 (7)	
O4	−0.03437 (19)	−0.05325 (16)	0.1499 (3)	0.0870 (7)	
O5	−0.11189 (19)	0.05489 (17)	0.1563 (4)	0.1005 (9)	
O6	0.0466 (2)	0.24889 (16)	0.5560 (5)	0.1221 (11)	
O7	0.19530 (19)	0.22737 (13)	0.7234 (4)	0.0908 (8)	
N1	0.40070 (16)	−0.01091 (13)	0.6539 (3)	0.0560 (6)	
H1	0.4020	0.0374	0.6654	0.067*	
N2	−0.0365 (2)	0.01221 (19)	0.2005 (3)	0.0721 (7)	
N3	0.1241 (2)	0.20962 (15)	0.6060 (4)	0.0752 (7)	
C1	0.5738 (2)	0.07809 (15)	0.8276 (3)	0.0540 (6)	
C2	0.57853 (19)	−0.00579 (14)	0.8223 (3)	0.0496 (6)	
C3	0.6705 (2)	−0.04275 (16)	0.9032 (4)	0.0585 (7)	
H3	0.7267	−0.0147	0.9634	0.070*	
C4	0.6794 (2)	−0.12038 (17)	0.8952 (4)	0.0691 (8)	
H4	0.7408	−0.1446	0.9502	0.083*	
C5	0.5968 (3)	−0.16112 (17)	0.8055 (5)	0.0763 (9)	
H5	0.6032	−0.2133	0.7988	0.092*	
C6	0.5043 (2)	−0.12685 (16)	0.7246 (4)	0.0676 (8)	
H6	0.4491	−0.1559	0.6648	0.081*	
C7	0.4936 (2)	−0.04870 (15)	0.7326 (3)	0.0524 (6)	
C8	0.3099 (2)	−0.03932 (16)	0.5632 (4)	0.0582 (7)	
C9	0.22294 (19)	0.01719 (15)	0.5044 (3)	0.0518 (6)	
C10	0.1393 (2)	−0.00719 (16)	0.3779 (3)	0.0560 (7)	
H10	0.1405	−0.0555	0.3293	0.067*	
C11	0.0544 (2)	0.04096 (18)	0.3248 (3)	0.0588 (7)	
C12	0.0482 (2)	0.11277 (17)	0.3938 (4)	0.0627 (7)	
H12	−0.0095	0.1446	0.3568	0.075*	
C13	0.1312 (2)	0.13480 (15)	0.5197 (4)	0.0587 (7)	
C14	0.2185 (2)	0.08975 (15)	0.5764 (4)	0.0545 (6)	
H14	0.2736	0.1073	0.6611	0.065*	
C15	0.6721 (3)	0.19029 (18)	0.9047 (6)	0.0866 (10)	
H15A	0.6505	0.2096	0.7838	0.104*	
H15B	0.6251	0.2119	0.9863	0.104*	
C16	0.7843 (3)	0.2102 (2)	0.9632 (9)	0.139 (2)	
H16A	0.7925	0.2640	0.9594	0.208*	
H16B	0.8302	0.1870	0.8841	0.208*	
H16C	0.8039	0.1926	1.0847	0.208*	

### Atomic displacement parameters (Å^2^)

**Table:**

	*U*^11^	*U*^22^	*U*^33^	*U*^12^	*U*^13^	*U*^23^
O1	0.0586 (11)	0.0578 (12)	0.0953 (15)	−0.0026 (9)	−0.0149 (10)	−0.0026 (10)
O2	0.0540 (11)	0.0594 (11)	0.0863 (14)	0.0043 (9)	−0.0075 (10)	0.0016 (10)
O3	0.0770 (14)	0.0613 (13)	0.1109 (18)	−0.0009 (11)	−0.0198 (13)	−0.0172 (12)
O4	0.0805 (15)	0.1026 (19)	0.0738 (15)	−0.0281 (14)	−0.0077 (11)	−0.0029 (13)
O5	0.0623 (14)	0.135 (2)	0.0963 (18)	0.0002 (14)	−0.0259 (13)	0.0126 (16)
O6	0.0978 (19)	0.101 (2)	0.158 (3)	0.0457 (16)	−0.0271 (18)	−0.0201 (18)
O7	0.0794 (15)	0.0655 (14)	0.122 (2)	−0.0015 (11)	−0.0130 (14)	−0.0100 (13)
N1	0.0480 (12)	0.0551 (12)	0.0622 (14)	0.0009 (9)	−0.0043 (10)	0.0008 (10)
N2	0.0546 (15)	0.105 (2)	0.0549 (14)	−0.0166 (15)	−0.0023 (11)	0.0122 (14)
N3	0.0634 (15)	0.0656 (15)	0.093 (2)	0.0088 (13)	−0.0045 (14)	0.0049 (14)
C1	0.0465 (14)	0.0586 (15)	0.0560 (15)	0.0011 (12)	0.0016 (11)	0.0001 (12)
C2	0.0458 (13)	0.0576 (15)	0.0452 (13)	0.0029 (11)	0.0045 (10)	0.0006 (11)
C3	0.0506 (15)	0.0693 (17)	0.0544 (15)	0.0061 (12)	0.0004 (11)	0.0038 (12)
C4	0.0638 (17)	0.0657 (18)	0.0753 (19)	0.0196 (14)	−0.0021 (15)	0.0069 (15)
C5	0.077 (2)	0.0551 (17)	0.094 (2)	0.0141 (15)	−0.0024 (17)	−0.0024 (16)
C6	0.0644 (17)	0.0556 (16)	0.080 (2)	0.0028 (13)	−0.0027 (14)	−0.0031 (14)
C7	0.0497 (14)	0.0573 (15)	0.0500 (14)	0.0061 (11)	0.0052 (11)	0.0035 (11)
C8	0.0514 (15)	0.0647 (17)	0.0573 (15)	−0.0025 (12)	0.0015 (12)	−0.0026 (13)
C9	0.0438 (13)	0.0612 (16)	0.0501 (14)	−0.0029 (11)	0.0038 (11)	0.0037 (12)
C10	0.0486 (14)	0.0701 (17)	0.0488 (14)	−0.0094 (12)	0.0034 (11)	0.0023 (12)
C11	0.0437 (14)	0.085 (2)	0.0463 (14)	−0.0120 (13)	0.0009 (11)	0.0105 (13)
C12	0.0464 (14)	0.0742 (19)	0.0664 (17)	0.0013 (13)	0.0016 (12)	0.0177 (15)
C13	0.0488 (14)	0.0588 (16)	0.0682 (17)	−0.0017 (12)	0.0053 (12)	0.0104 (13)
C14	0.0458 (13)	0.0598 (15)	0.0565 (15)	−0.0060 (11)	−0.0001 (11)	0.0051 (12)
C15	0.075 (2)	0.0586 (18)	0.121 (3)	−0.0012 (16)	−0.011 (2)	−0.0072 (18)
C16	0.086 (3)	0.075 (3)	0.243 (6)	−0.011 (2)	−0.036 (3)	−0.007 (3)

### Geometric parameters (Å, °)

**Table:**

O1—C1	1.319 (3)	C5—C6	1.378 (4)
O1—C15	1.460 (4)	C5—H5	0.9300
O2—C1	1.217 (3)	C6—C7	1.393 (4)
O3—C8	1.225 (3)	C6—H6	0.9300
O4—N2	1.221 (4)	C8—C9	1.503 (4)
O5—N2	1.221 (4)	C9—C10	1.390 (4)
O6—N3	1.214 (3)	C9—C14	1.396 (4)
O7—N3	1.212 (3)	C10—C11	1.381 (4)
N1—C8	1.346 (3)	C10—H10	0.9300
N1—C7	1.405 (3)	C11—C12	1.378 (4)
N1—H1	0.8600	C12—C13	1.370 (4)
N2—C11	1.468 (3)	C12—H12	0.9300
N3—C13	1.480 (4)	C13—C14	1.376 (4)
C1—C2	1.488 (4)	C14—H14	0.9300
C2—C3	1.395 (3)	C15—C16	1.458 (5)
C2—C7	1.407 (4)	C15—H15A	0.9700
C3—C4	1.382 (4)	C15—H15B	0.9700
C3—H3	0.9300	C16—H16A	0.9600
C4—C5	1.366 (4)	C16—H16B	0.9600
C4—H4	0.9300	C16—H16C	0.9600
			
C1—O1—C15	117.0 (2)	O3—C8—C9	119.6 (2)
C8—N1—C7	129.4 (2)	N1—C8—C9	115.6 (2)
C8—N1—H1	115.3	C10—C9—C14	119.1 (2)
C7—N1—H1	115.3	C10—C9—C8	116.7 (2)
O5—N2—O4	123.3 (3)	C14—C9—C8	124.1 (2)
O5—N2—C11	117.9 (3)	C11—C10—C9	119.4 (3)
O4—N2—C11	118.7 (3)	C11—C10—H10	120.3
O7—N3—O6	124.2 (3)	C9—C10—H10	120.3
O7—N3—C13	118.0 (2)	C12—C11—C10	122.6 (2)
O6—N3—C13	117.8 (3)	C12—C11—N2	118.8 (3)
O2—C1—O1	122.5 (2)	C10—C11—N2	118.4 (3)
O2—C1—C2	125.2 (2)	C13—C12—C11	116.6 (3)
O1—C1—C2	112.3 (2)	C13—C12—H12	121.7
C3—C2—C7	119.1 (2)	C11—C12—H12	121.7
C3—C2—C1	119.4 (2)	C12—C13—C14	123.5 (3)
C7—C2—C1	121.5 (2)	C12—C13—N3	118.2 (2)
C4—C3—C2	121.0 (3)	C14—C13—N3	118.2 (2)
C4—C3—H3	119.5	C13—C14—C9	118.8 (2)
C2—C3—H3	119.5	C13—C14—H14	120.6
C5—C4—C3	119.2 (3)	C9—C14—H14	120.6
C5—C4—H4	120.4	C16—C15—O1	107.6 (3)
C3—C4—H4	120.4	C16—C15—H15A	110.2
C4—C5—C6	121.7 (3)	O1—C15—H15A	110.2
C4—C5—H5	119.2	C16—C15—H15B	110.2
C6—C5—H5	119.2	O1—C15—H15B	110.2
C5—C6—C7	119.9 (3)	H15A—C15—H15B	108.5
C5—C6—H6	120.0	C15—C16—H16A	109.5
C7—C6—H6	120.0	C15—C16—H16B	109.5
C6—C7—C2	119.1 (2)	H16A—C16—H16B	109.5
C6—C7—N1	122.3 (2)	C15—C16—H16C	109.5
C2—C7—N1	118.6 (2)	H16A—C16—H16C	109.5
O3—C8—N1	124.8 (3)	H16B—C16—H16C	109.5

### Hydrogen-bond geometry (Å, °)

**Table:**

*D*—H···*A*	*D*—H	H···*A*	*D*···*A*	*D*—H···*A*
N1—H1···O2	0.86	1.92	2.641 (3)	140.
C16—H16A···O3^i^	0.96	2.55	3.402 (4)	148

Symmetry codes: (i) −*x*+1, *y*+1/2, −*z*+3/2.
